# Improved adaptive-phase fuzzy high utility pattern mining algorithm based on tree-list structure for intelligent decision systems

**DOI:** 10.1038/s41598-023-50375-y

**Published:** 2024-01-10

**Authors:** Jing Chen, Aijun Liu, Hongjun Zhang, Shengyi Yang, Hui Zheng, Ning Zhou, Peng Li

**Affiliations:** 1 School of Internet of Things, Nanjing University of Posts and Telecommunications, Nanjing, 210023 China; 2 Baotou Teachers’ College of Inner Mongolia University of Science and Technology, Baotou, 014030 Inner Mongolia China; 3 School of Physics and Mechatronic Engineering, Guizhou Minzu University, Guiyang, 550025 China; 4 Software and Computational Systems Program, Data 61, CSIRO, Canberra, Australia; 5 School of Computer Science, Nanjing University of Posts and Telecommunications, Nanjing, 210023 China; 6 Institute of Network Security and Trusted Computing, Nanjing, 210023 China

**Keywords:** Computer science, Information technology

## Abstract

With the rapid development of AI and big data mining technologies, computerized medical decision-making has become increasingly prominent. The aim of high-utility pattern mining (HUPM) is to discover meaningful patterns in medical databases that contribute to maximizing the utility from the perspective of diagnosis. However, HUPM pays less attention to the interpretability and explainability of these patterns in medical decision-making scenarios. This paper proposes a novel algorithm called the Improved fuzzy high-utility pattern mining (IF-HUPM) to address this problem. First, the paper applies a fuzzy preprocessing method to divide the fuzzy intervals of a medical quantitative data set, which enhances the fuzziness and interpretability of the data. Next, in the process of IF-HUPM, both fuzzy tree and list structures are employed to calculate fuzzy high-utility values. By combining the characteristics of the one-stage and two-stage algorithms of HUPM, an adaptive-phase Fuzzy HUPM hybrid frame is proposed. The experimental results demonstrate that the proposed IF-HUPM algorithm enhances both accuracy and efficiency and the mining process requires less time and space on average.

## Introduction

In the era of AI and the interpretation of medical diagnostic decisions, the use of current computer-based medical diagnostic and predictive methods^1^ can effectively assist doctors in the accuracy of medical diagnosis. It is also necessary to analyse the excavation results in terms of interpretation, which is convenient for decision-makers to interpret in their decision-making, recommendation system^2–5^, and Internet of things^6,7^.

Extracting valuable information^8,9^ and knowledge from large datasets^10–12^ requires the use of various approaches such as statistics, machine learning, artificial intelligence, and database technology to find patterns, associations^13–16^, and rules in diverse areas^17–19^. Zadeh^20^ utilized association rule mining methods to explore potential drugs or drug combinations associated with newly diagnosed diabetes. Similarly, Vhaduri et al.^21^ conducted a study where they provided continuous glucose monitoring devices to 44 participants to investigate the application n diabetes management.

While traditional Apriori algorithm^22^ and Frequent Pattern Mining (FPM) algorithms^23^ solely focus on identifying the existence of item sets, disregarding their actual value, a classic technique known as High-Utility Item set Mining (HUIM) addresses this limitation. Liu et al.^24^ introduced HUIM in 2005, which incorporates both the frequency of occurrence and the value contained within item sets. It achieves this by utilizing external utilities and internal utilities. For instance, by analyzing a large amount of data from diabetes patients, HUPM may reveal significant associations between specific physiological indicators, other factors, and the severity of diabetes risk. These patterns offer valuable reference information for doctors, aiding them in better-assessing patients' conditions and developing personalized treatment plans^25,26^.

However, HUPM is primarily applied to mine databases containing profit information^27–30^, without considering the interpretability and explainability of these items. Many researchers have conducted relevant studies to address this issue. In 2016, Fournier-Viger proposed FHMUF algorithm that combines HUPM with fuzzy set theory to mine Fuzzy High-Utility Pattern (FHUP). To identify FHUPs, the method preprocesses and calculates fuzzy support and fuzzy utility values. For mining FHUPs, it is based on fuzzy partitioning and multilevel fuzzy set theory. The rule-based fuzzy set theory may effectively soften the threshold and border in the medical environment, alleviating the problem of sharp points. Researchers have proposed many efficient usable itemset mining algorithms, but the resultant itemsets only show the correlation between efficient usable items, and the efficient usable itemset mining is still mainly applied to mining databases with profit information without considering the numerical information of these items, which has the problem of poor interpretability.

To optimize computerized medical decision-making—a field traditionally underserved by existing HUPM, we propose an novel improved fuzzy high utility pattern mining algorithm (IF-HUPM) with adaptive-phase strategy based on Tree-List Structure under medical senoras for decision making. The proposed IF-HUPM algorithm integrates fuzzy preprocessing to bolster interpretability of quantitative medical databases, thereby refining diagnostic accuracy. It also merges fuzzy tree and list structures, and marries one-phase and two-phase algorithmic properties of HUPM into an adaptive, hybrid framework, further enhancing mining efficiency. Experimental results highlight that IF-HUPM not only elevates mining accuracy but economizes on time and space—critical when dealing with extensive real-time medical data. In essence, IF-HUPM provides a viable solution to the prevalent challenge of pattern interpretability in medical decision-making, thus marking a significant stride towards the enhancement of intelligent decision-making systems and medical service quality. The three main contributions of this paper are as follows:

1. To address the issues of sharp peaks in data and poor interpretability in existing efficient itemset mining models, we introduce the fuzzy sets theory to FHUPM. We proposed IF-HUPM algorithm which can adapt to diverse data properties in various medical contexts which is efficient in terms of space–time complexity and has good performance, making it suitable for practical use in medical data analysis.
2. In this paper, we propose the Adaptive-Phase Fuzzy UFH Hybrid Frame for fuzzy high-utility pattern mining based on multi-dimensional data fuzzification. To achieve this, we employ a switch module that combines one-stage and two-stage mining methods. This approach allows us to develop an interpretable decision-making framework for medical databases, which is efficient and effective when applied to diverse data properties.
3. Experimental results on multiple publicly available real-world datasets demonstrate that introducing fuzziness can better reflect the meaning of the numerical attributes of the data, and enhance the interpretability and comprehensibility of the mining results.

## Related work on fuzzy high-utility pattern mining (FHUPM)

The fuzzy data mining method is a common technique in data mining. Zadeh et al.^20^ proposed the membership function as the feature function of fuzzy sets, which is the core of fuzzy sets, in his paper Fuzzy Sets, published in 1965. As the application scope of fuzzy sets expands, some extended fuzzy sets, such as interval-valued fuzzy sets, intuitive fuzzy sets, Vague sets, type II fuzzy sets, etc.^31^, are gradually adopted in various fields.

In 2009, Wang et al.^32^ proposed a fuzzy approach for mining high-utility quantitative itemsets. Their method considers both profits and quantities of items to discover FHUIs from quantitative databases. Fournier-Viger et al.^33^ provided an up-to-date survey that serves as an introduction and guide to recent advances and opportunities in the field of mining high-utility item sets (HUIs) in 2017. Mohbey introduced a new approach called HFUBPM (High Fuzzy Utility Based Patterns Mining)^34^ for extracting high fuzzy utility patterns from mobile web services access sequences. They designed a modified tree structure based on frequent-pattern trees and propose a mining algorithm to extract high temporal fuzzy utility patterns from transactional datasets with temporal properties.

Hong et al. have noted that the aforementioned approaches can only handle binary situations, which complicates the representation of discovered knowledge as linguistic variables^35^. A study by Wu et al. investigate a genetic algorithm-based framework for mining high fuzzy utility itemsets^36^. Previous research has primarily focused on finding high fuzzy utility itemsets in small databases, a circumstance which is not reflective of real-world scenarios in big data environments. In 2022, Ganesan et al.^37^ conducted an investigation into HUFPM, specifically aiming to discover product sets with high benefits.

Gan et al.^38^ propose a method called Pattern Growth Fuzzy Utility Mining (PGFUM), which leverages fuzzy set theory for mining fuzzy high-utility sequences with linguistic meaning. In 2022, Wan et al. propose a novel one-phase temporal fuzzy utility item set mining approach called TFUM^39^. Ryu et al.^40^ propose a scalable and efficient approach with a novel data structure for mining high temporal fuzzy utility patterns without generating candidates. All existing HUSRM algorithms aim to find high-utility partially-ordered sequential rules (HUSRs), which are not consistent with reality and may generate fake HUSRs.

To address this, Zhang et al.^41^ propose two novel algorithms, called TotalSR and TotalSR + , which aim to identify all High-Utility Totally-ordered Sequential Rules (HTSRs). However, due to the symmetry of frequent itemset pattern (FIPs), these strategies cannot be directly applied to Periodic High-Utility Sequential Pattern Mining (PHUSPM). To address these issues, Dinh et al.^42^ propose the Stable Periodic High-Utility Sequential Pattern Mining (HUSPM) algorithm.

To reduce complexity and obtain a compact set of frequent high-utility sequential patterns^43^, Huang et al. proposed an efficient algorithm called TMKU based on the TargetUM algorithm to discover the top-k Target High-Utility Itemsets (top-k THUIs) in 2023. The research achievements and latest algorithms^44^ mentioned have led to significant advancements in FHUPM algorithms, including algorithm innovation, improvement of data structures, and performance enhancement.

However, there are limitations in the research and applicability of these algorithms in multidimensional medical data and real healthcare scenarios. Mining data in the medical field, with multidimensional data and complex features, presents a challenging task. By conducting in-depth research on existing algorithms and better adapting them to the needs of the medical field, it is possible to improve the accuracy and interpretability of mining results.

In this paper, we addresses the challenges of FHUPM in medical data mining. Specifically, we leverage the characteristics of one-phase and two-phase mining. This approach aims to enhance the effectiveness, applicability, and interpretability of the method in medical scenarios. Additionally, integrating the characteristics of one-phase and two-phase mining can help reduce computational complexity and improve mining efficiency. This methodology could possibly enable more efficient mining processes of large-scale medical data and facilitate a better exploration of patterns and associations within the data.

## FHUPM algorithm and framework

This section focuses on the application of fuzzy set theory to the process of fuzzifying medical databases, followed by the mining of their FHUP^45–49^.

### Hybrid high utility framework

HUPM algorithms predominantly employ either one-phase or two-phase approaches for the discovery of High Utility Itemsets (HUIs). In the case of two-phase algorithms, a tree-based data structure is commonly employed for the generation of candidate itemsets and their subsequent validation. This data structure facilitates the storage of upper-bound utility estimations for each itemset at each node, enabling the rapid generation of candidate HUIs. However, the generation of candidate HUIs continues to represent the primary computational overhead in two-phase mining algorithms.

**Figure 1:**
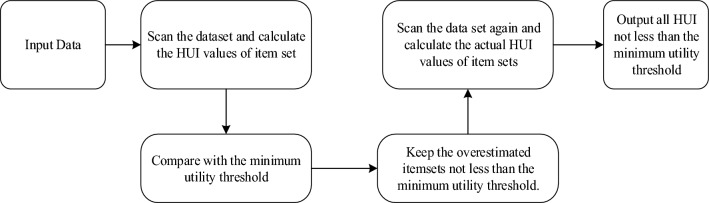
Two-phase HUIM model.

Conversely, one-phase algorithms typically leverage inverted lists and transaction projection methodologies, thereby circumventing the need for candidate item set generation and enabling precise utility value calculations. The two-phase HUIM model is depicted in Fig. 1 below.

To combine the advantages of one-phase and two-phase algorithm frameworks, Dawar et al.^50^ proposed a hybrid algorithm in 2017 by integrating UP-Growth + and FHM. This algorithm essentially functions as a hybrid framework, integrating both one-phase and two-phase approaches. In various scenarios, it allows for the dynamic selection of an algorithm framework, or executing a portion of one framework before transitioning to the algorithm of another framework. However, a significant challenge lies in defining the criteria for this transition and determining the most appropriate method for implementing the switch.

### Basic definitions

For the original database in the medical field, we need to apply fuzzy set theory to fuzzify each transaction and obtain the fuzzy value of each item set. This not only solves the problem of sharp point division but also generates the internal utility value of the database based on the fuzzy value. For instance, in a specific transaction, the fasting blood glucose value is 7. Its membership degree to the hyperglycemia interval is 1, and its internal utility value after processing is 12. The data can then be mined for fuzzy but effective usage patterns. However, in the process of mining, most algorithms either use one-stage mining to construct a utility list and calculate the actual utility value, or use a two-stage construction tree to first calculate the overestimated utility value and then the actual utility value. In this paper, a hybrid framework will be used to integrate a one-stage algorithm and a second-order feature. This integration will enable the mining algorithm to perform effectively on data sets with diverse characteristics. Before describing the fuzzification process and mining algorithm in detail, we will first explain and define their symbolic meanings. First, each transaction contains a group of items and is associated with a unique transaction identifier, Tid. Let I = {i1, i2, i3, …, in} be the complete set of distinct items that appear in the transaction database.

As shown in Table 1, the transaction data set contains six transactions $T_{j} = \left\{ {T_{1} ,T_{2} ,T_{3} ...T_{6} } \right\}$ and eight items I = A, B, C, D, E, F, G, and H. The internal utility of item X in a transaction $T_{j} \in \left\{ {T_{1} ,T_{2} ,T_{3} ...T_{6} } \right\}$ is defined as the number of x in a transaction ${\text{T}}_{{\text{j}}}$, expressed as ${\text{IU}}\left( {{\text{x,T}}_{{\text{j}}} } \right)$, and the external utility of item X is expressed as ${\text{EU}}\left( {\text{x}} \right)$. As shown in Table 1, the internal utility of item A in the transaction ${\text{T}}_{{1}}$ is. As shown in Table 2, the external utility values of items A, B, C, D, E, F, G, and H are 5, 2, 1, 2, 3, 5, 1, 1.

**Table 1:** Transaction database.

TID	Transaction	Transaction utility
1	(A: 1) (C: 10) (D: 1)	17
2	(B: 2) (C: 4) (E: 1) (G: 2)	13
3	(A: 2) (B: 2) (D: 6) (E: 2) (F: 1)	37
4	(B: 4) (C: 13) (D: 3) (E: 1)	30
5	(A: 2) (C: 6) (E: 2) (G: 5)	27
6	(A: 6) (B: 1) (C: 1) (D: 4) (H: 2)	43

**Table 2:** Utility list.

Item	A	B	C	D	E	F	G	H
External utility value	5	2	1	2	3	5	1	1

#### Definition 1

^45^. Item's utility in a transaction. The utility of item *X* in transaction $T_{j}$ is expressed as ${\text{U}}\left( {x{\text{,T}}_{{\text{j}}} } \right)$, and defined as

1 $$ U\left( {x,T{}_{j}} \right) = IU\left( {x,T_{j} } \right) \times EU\left( x \right) $$

#### Definition 2

^46^. The utility of an item set in a transaction. The utility of item set *X* in transaction $T_{j}$ is expressed as $U\left( {x,T_{j} } \right)$ and defined as

2 $$ U\left( {X,T_{j} } \right) = \sum\nolimits_{x \in X} {U\left( {x,T_{j} } \right)} $$

#### Definition 3

^47^. Utility of item sets in a transaction database. The utility value of item set *X* in the transaction database is expressed as $U\left( X \right)$, is the sum of the utility values of item set *X* in all database transactions, and is defined as

3 $$ U\left( X \right) = \sum\limits_{{x \subseteq T_{j \wedge } T_{j} \in D}} {U\left( {X,T_{j} } \right)} $$

#### Definition 4

^48^. Transaction utility. The transaction utility of transaction $T_{j}$ is expressed as $TU\left( {T_{j} } \right)$, where m is the number of items of transaction $T_{j}$, and defined as

4 $$ TU\left( {T_{j} } \right) = \sum\nolimits_{i}^{m} {U\left( {x_{i} ,T_{j} } \right)} $$

In Tables 1 and 2. Transaction utility of n $T_{5}$ is expressed as $TU\left( {T_{5} } \right) = 2 \times 5 + 6 \times 1 + 2 \times 3 + 5 \times 1 = 27$.

#### Definition 5

^49^. The support of item set. The support of item set *X* is defined as the number of transactions containing *X* in data set, expressed as ${\text{support}}(X)$. As shown in Table 1, support of item set $\{ A,C,D\}$ is 2 and appears in transactions $T_{1}$ and $T_{6}$.

#### Definition 6

Transaction weighted utility. The transaction weighted utility of item set *X* is the sum of all transaction utility of item set *X*, expressed as $TWU\left( X \right)$. For example the value of $TWU\left( G \right)$ is $TWU\left( G \right) = TU\left( {T_{2} } \right) + TU\left( {T_{5} } \right) = 13 + 27 = 40$.

#### Definition 7

High-utility value. Suppose the utility value of item set *X* in the transaction database is not less than the minimum utility threshold min-utility set by the user. In that case, the high-utility value is the HUIs. For any item set *X*, if the TWU of *X* is less than the minimum utility threshold min-utility, then any super-set of X must not a HUI.

As shown in Table 1, according to Eq. (4) the transaction utility of A is 124. If min-utility is 120, then the super-set of item A has the possibility of high-utility item sets. If min-utility is 160, the super-set of item A must not be an HUI.

#### Definition 8

Fuzzy set is an extension of the clear set in the traditional set members will only belong to a certain set. In fuzzy sets, members can belong to multiple sets simultaneously. The membership function of the traditional clear set is in Eq. (5).

5 $$ {\text{U}}_{{\text{A}}} ({\text{X}}) = \left\{ {\begin{array}{*{20}c} 1 & {{\text{if X }} \in A} \\ 0 & {{\text{if X}} \notin A} \\ \end{array} } \right. $$

The fuzzy set theory extends this concept using tiny partial membership, ranging from 0 to 1. The membership function is formally defined in Eq. (6).

6 $$ {\text{U}}_{{\text{A}}} ({\text{X)}}:\,\,X \to \left[ {0,1} \right] $$

### Fuzzy high utility itemset construction

In the conventional medical scene, data acquired for various indicators typically manifest as floating-point values. Both the classical FIM algorithm and the HUIM algorithm excel in processing Boolean, binary, and other data types. They can unveil relationships among different data features, thereby presenting distinct advantages in disease prediction and diagnosis, exerting a unique impact on the future evolution of diagnostic practices. In a conventional medical setting, the data obtained from indicators are predominantly in the form of floating-point values. To address this, after processing raw data and handling inconsistencies, various data features undergo fuzzification based on reference values for human indicators. Subsequently, they are transformed into Boolean, binary, or categorical data, and data mining is executed using mining algorithms^1,2,9,17^. The direct partition interval method and the fuzzy partition interval method are commonly employed to convert floating-point data from physical examinations into Boolean data. The former is straightforward but may lead to the issue of sharp boundaries in certain situations. Consider, for instance, the measurement of fasting blood glucose levels in diabetes screenings during a routine medical checkup.

The classification of a person whose fasting glucose level is 6.4 mmol/L can be approached using the fuzzy partition interval method. This method helps avoid sharp distinctions by extending the data space into the fuzzy space and dividing it into intervals. By assigning membership degrees, a floating-point number between 0 and 1 to each interval, we can assess the likelihood of different classifications.

In the case of a fasting blood glucose level of 6.4 mmol/L, we can assign membership degrees to different categories. For example, it can be classified as hyperglycemia with a high membership degree (e.g., 0.99) if the threshold for hyperglycemia is set below 6.4 mmol/L. On the other hand, if the threshold for normal blood glucose is set above 6.4 mmol/L, it can be classified as normal blood glucose with a low membership degree (e.g., 0.05). Additionally, if the threshold for hypoglycemia is set significantly lower than 6.4 mmol/L, it can be classified as hypoglycemia with a very low membership degree (e.g., 0).

By using this approach, we can capture the uncertainty and gradual transition between different classifications rather than relying solely on a fixed threshold. It allows for a more nuanced interpretation of blood glucose levels and helps in identifying potential health conditions that may require further examination.

**Figure 2:**
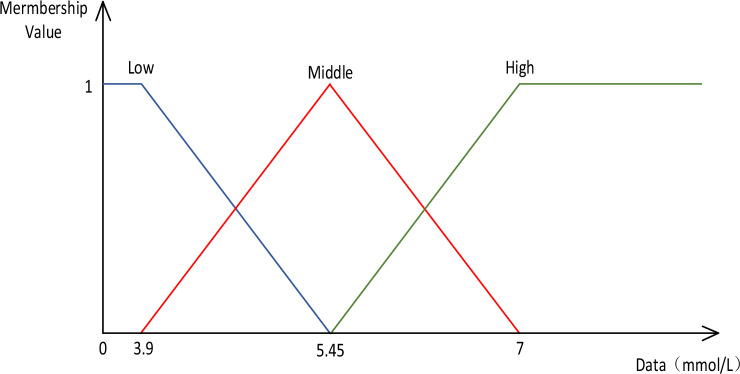
Fuzzy membership function for fasting blood glucose.

This paper proposes a fuzzy and efficient pattern mining algorithm for medical data. The algorithm aims to de-weight the diabetes dataset, fill in missing values, and normalize the data. The normalization process ensures that attribute values, such as fast blood glucose, fall within the range of 0–7. This range is convenient for fuzzy processing. The algorithm takes into account the medical reference values of various indicators for human beings, as shown in Table 3. In Table 3, we outline the reference standards for human medical parameters. Based on Table 3, we first process the original data by normalizing it according to the Fuzzy Membership Function shown in Fig. 2. Table 4 presents the original data set from the Pima Indians data set^44^, where the order of attributes represented by the data from left to right corresponds to the order of attributes in Table 3. On the right side of Table 4, 1 represents diabetic patients and 0 represents non-diabetic patients. Following fuzzification, the data set is partitioned into three sections, as shown in Table 5 below, where Items represent physical examination attributes. This transaction's item utilities represent the membership value of each attribute within the fuzzy interval. As we are more concerned with the health evaluation of physical examinations, the external utility of other attributes in the external utility table is set to 1 by default. In contrast, the external utility without abnormality in the health evaluation is set to 0, reducing its utility value and focusing on the abnormal situations we are concerned about. Transaction utility represents the total number of attributes that belong to the fuzzy interval, which is the sum of the values in the second and third columns.

**Table 3:** Reference table of medical monitoring of human parameters.

Item tag	Items of human parameters	Reference range
1	BMI (body mass index)	16–30 kg/m^2^
2	fasting blood glucose	3.2–6.5 mmol/L
3	glycosylated hemoglobin	6.1–7.9% mmol/L
4	Serum alanine aminotransferase	9–50 U/L
5	serum glutamic oxalacetic transaminase	10–40 U/L
6	Blood protein	37–55 g/L
7	Total bilirubin	3.4–28 μmol/L
8	Serum creatinine	50–111 μmol/L
9	Blood urea nitrogen	3.1–8.0 mmol/L
10	Total cholesterol	2.8–5.6 mmol/L
11	Triglyceride	0.45–1.69 mmol/L
12	Serum low-density lipoprotein cholesterol	1.03–1.61 mmol/L
13	Serum high-density lipoprotein cholesterol	1.04–1.96 mmol/L
14	Health assessment	16–30

**Table 4:** Original data sets of medical monitoring of human parameters.

TID	Data sets	Index of health
1	24.97, 5.2, 126, 32, 31, 37, 15.7, 73.4, 3.7, 2.7, 1, 2.3, 1.15	1
2	25.11, 5.8, 102, 9, 12, 42, 6.3, 59, 4.7, 2.9, 0.9, 1.09, 1	1
3	25.82, 4.9, 124, 23, 31, 37, 11.6, 65, 5.6, 5.07, 1.3, 2.06, 1.62	1
4	24.56, 5.4, 141, 35, 34, 45, 6.5, 52 7, 4.1, 1.8, 3.24, 1.62	1
5	24.24, 5.4, 140, 17, 18, 44, 13.3, 68, 8, 5.1, 1.2, 3.29, 1.22	1
6	23.59, 4.7, 127, 24, 34, 43, 7.3, 120, 4.8, 3.7, 1.9, 2.3, 1.2	0
7	23.44, 6.5, 136, 33, 30, 43, 3.4, 88, 3.5, 5.31, 1.7, 2.67, 1.32	1
8	20.44, 5.6, 120, 12, 25, 37, 12, 50, 8, 5.6, 1.2, 2.3, 1.3	0

**Table 5:** Fuzzification of original data sets of medical monitoring of human parameters.

TID	Items tags of medical monitoring of human parameters	Transaction utility	Item utilities for this transaction
1	1 2 3 4 5 6 7 8 9 10 11 12 13 14	75	7 7 10 9 5 0 10 4 1 0 6 4 2 10
2	1 2 3 4 5 6 7 8 9 10 11 12 13 14	46	6 3 2 0 0 4 1 9 6 0 5 0 0 10
3	1 2 3 4 5 6 7 8 9 10 11 12 13 14	93	5 10 9 6 5 0 6 7 10 8 9 2 6 10
4	1 2 3 4 5 6 7 8 9 10 11 12 13 14	86	7 6 5 7 3 7 1 10 3 4 7 10 6 10
5	1 2 3 4 5 6 7 8 9 10 11 12 13 14	86	8 6 5 3 4 6 8 6 0 8 8 10 4 10
6	1 2 3 4 5 6 7 8 9 10 11 12 13 14	66	9 9 10 7 3 5 2 0 6 2 6 4 3 0
7	1 2 3 4 5 6 7 8 9 10 11 12 13 14	73	9 0 6 8 6 5 0 0 0 9 7 7 6 10
8	1 2 3 4 5 6 7 8 9 10 11 12 13 14	63	6 5 8 0 10 0 6 0 0 10 8 4 6 0

## The proposed algorithm

### Improved fuzzy high-utility pattern mining algorithm

This section aims to provide guidance on extracting high utility patterns from fuzzy databases. To achieve this goal, an improved fuzzy high-utility pattern mining algorithm with an adaptive-phase UFH hybrid frame is proposed. It is important to note that there is no clear winner between one-stage and two-stage mining algorithms, as their performance depends on the specific scenario. Thus, defining and implementing switching criteria can be a challenging task.

In this study, we propose the adaptive-phase fuzzy UFH hybrid framework to extract high-utility itemsets (HUIs) after the multi-dimensional data fuzzification. This algorithm works by first scanning the dataset and constructing a fuzzy tree after fuzzification. It then calculates the overestimated utility value and compares it to the lowest utility value in order to eliminate a portion of the itemset. Next, the actual utility value is calculated and compared to the minimum utility threshold value in order to identify the most relevant HUIs for the problem.

A single-stage mining algorithm is utilized. Scan the database for information using lists. Similar to the Apriori principle of generating item sets of varying lengths, this method omits the step of generating candidate itemsets and calculates real utility values directly. The candidate HUI is screened before constructing the utility tree. Using a list-based algorithm, the utility values are computed. Execute the tree-based algorithm first and switch to the list-based algorithm at a certain point.

**Figure 3:**
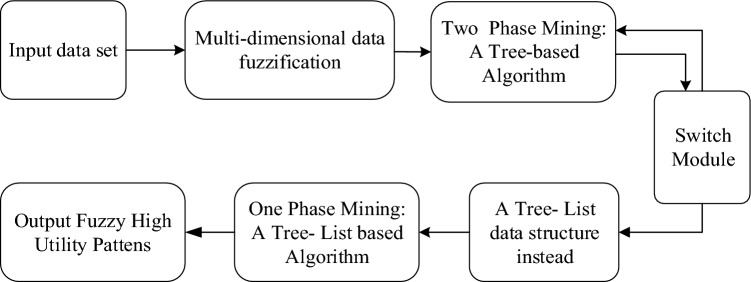
Fuzzy UFH hybrid frame with adaptive phase.

The switching module can define switching criteria to assist in determining the optimal switching point to maximize overall efficiency. For example, the switching criteria can be determined by observing the data distribution. The Fuzzy UFH hybrid framework with adaptive-phase is illustrated in Fig. 3.

**Figure 4:**
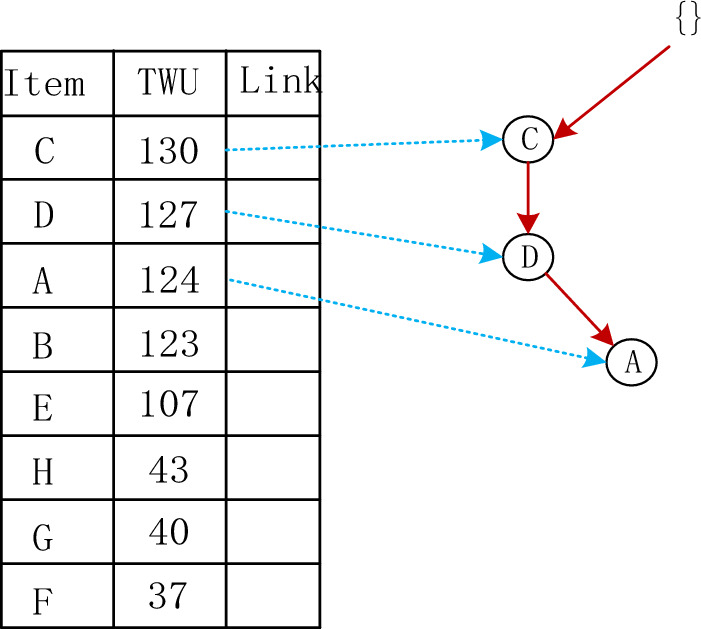
The process of building UP-tree (TID = 1).

**Figure 5:**
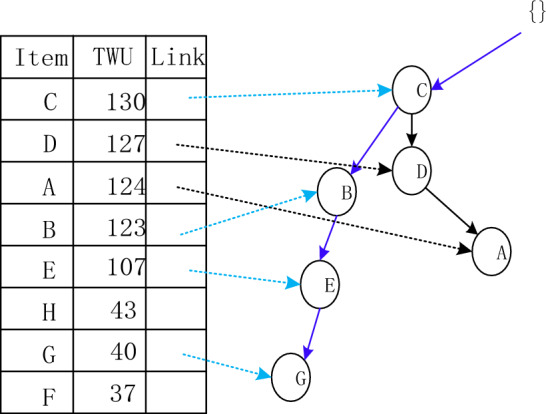
The process of building UP-tree (TID = 2).

**Figure 6:**
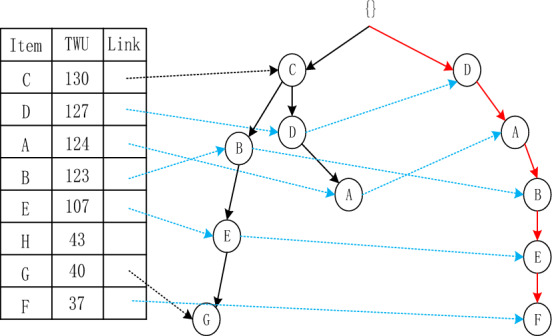
The process of building UP-tree (TID = 3).

**Figure 7:**
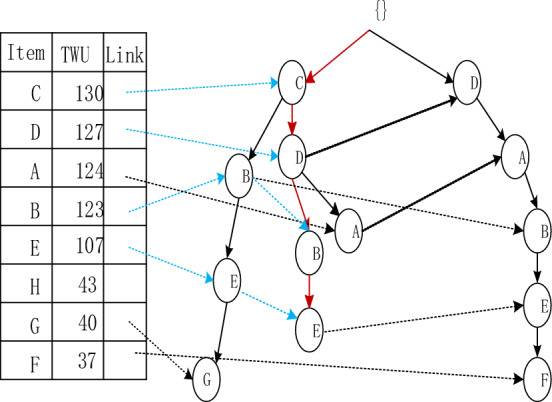
The process of building UP-tree (TID = 4).

**Figure 8:**
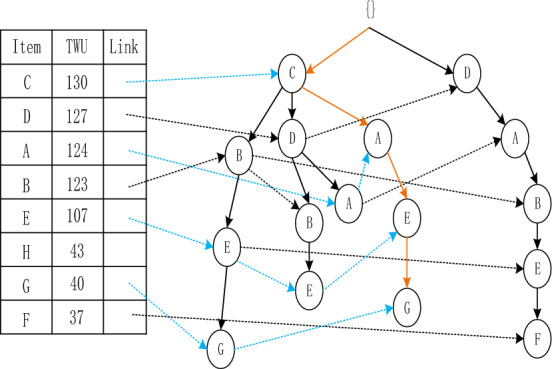
The process of building UP-tree (TID = 5).

**Figure 9:**
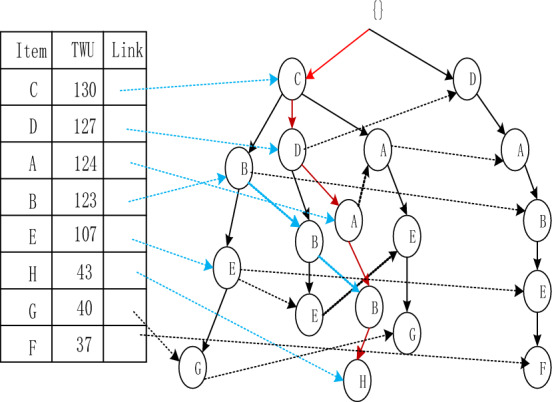
The process of building UP-tree (TID = 6).

The Adaptive-Phase UFH Hybrid Framework has the capability of incorporating both one-stage and two-stage mining algorithms. Two notable algorithms that utilize this framework are the two-stage recursive mining algorithm Up-growth, proposed by Tseng et al.^51^, and the vertical data mining algorithm proposed by Fournier-Viger et al.^52^. The mining process is introduced using the utility list data structure to mine HUI. To mine high-utility itemsets (HUI), the mining process employs the utility list data structure. Initially, the dataset is fuzzified and a fuzzy database is generated, which includes an internal utility table obtained from the initial database scan. Given the characteristics of the fuzzy database, the UFH algorithm constructs an Up-Tree and calculates the overestimated utility value, which is then used to eliminate high-utility candidate itemsets. Each node in the Up-Tree comprises the item name N, its overestimated utility value N.nu, the degree of support N.mount, a pointer n.parent node, and a pointer n.link to other nodes shown in Figs. 4 and 5. The root node is an empty node that points to the child node. A prefix itemset is an itemset formed by the path from the root node to any other node in the tree depicted in Figs. 6, 7, 8 and 9.

**Figure 10:**
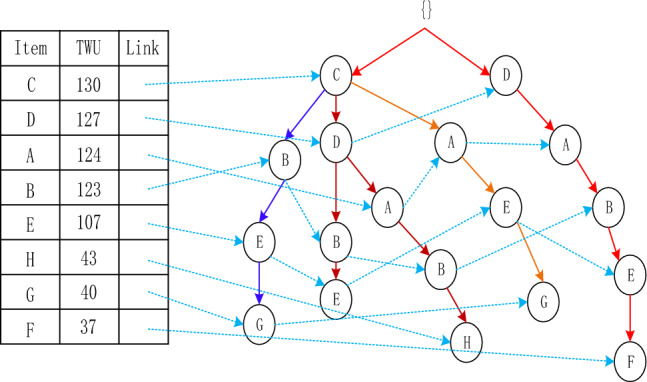
The whole process of UP-tree building.

Furthermore, a header table is maintained, which includes the name of the item set, its TWU value, and a pointer to the Up-Tree. The Up-Tree's nodes are arranged in descending order, based on the TWU values in the header table. To connect identical items from different transactions, they are linked using a linked list.The Up-Growth + algorithm calculates the transaction weighted utility (TWU) for all items during the initial database scan. Entries that have a TWU below the predetermined minimum utility threshold are removed from the database. TWU values in the header table are utilized to sort the database items within a transaction. Next, the new database's data is utilized to establish a UP-Tree. Figures 4, 5, 6, 7, 8 and 9 depict the up-tree process created using data from the provided sample database and Fig. 10 shows the whole process of UP-Tree building.

### Fuzzy hybrid mining (FHM) algorithm

In the proposed IF-FUPM algorithm, a Fuzzy Hybrid Mining model was applied for mining fuzzy high-utility itemsets based on the UFH algorithm (Hybrid Algorithm Based on UP-Growth + and FHM)^3^. FHM is a vertical data mining algorithm that utilizes the Utility-list data structure to extract high-utility itemsets. The Utility-list is a compact data structure designed to store the itemsets and their utility values that appear in a database.

FHM uses a utility-list to identify HUP. Utility-list is a compact data structure designed to store transaction database item sets and Utility values. It consists of three main columns: *TID *(transaction ID), *Iutils *(items utilities), and *Rutils *(residue utilities). TID is the unique identifier for each piece of transaction data stored in the transaction database. *Iutils* is the actual utility value of item set I in the transaction. *Rutils* is the residual utility of item set I.

Each database transaction's internal items are arranged and reorganized based on the ascending TWU value of an item set. Reorganized database is the name of the reorganized database. Table 6 illustrates a sample database transaction for reorganizing the database of Table 1. The utility list number structure for item sets {A} and {B} is depicted in Figs. 7 and 8. For example, in T1 transaction, the *Iutils* value of A is the product of internal utility value and external utility value of {A} in T1 is 12, in Eq. (7).

7 $$ Rutils\left( \{ {\text{A} \} ,T}_{{1}}  \right) = {\text{I}}utils\left( {\{ D\} ,{\text{T}}_{1} } \right) + {\text{I}}utils\left( {\{ C\} ,{\text{T}}_{1} } \right) $$

**Table 6:** The transaction database.

TID	Reference range	Transactional utility
1	(A: 1) (D: 1) (C: 10)	17
2	(G: 2) (E: 1) (B: 2) (C: 4)	13
3	(F: 1) (E: 2) (B: 2) (A: 2) (D: 6)	37
4	(E: 1) (B: 4) (D: 3) (C: 13)	30
5	(G: 5) (E: 2) (A: 2) (C: 6)	27
6	(H: 2) (B: 1) (A: 6) (D: 4) (C: 1)	43

In transaction T5, the *Iutils* value of A is the product of internal and external utility values of {A} in T5 database, namely $Iutils\left( {\{ {\text{A}\} ,T}_{{5}} } \right) = 2*5 = 10$, The *Rutils* value of A is the sum of the internal and external utility values of the {C} item set after the {A} item set after the transaction sorting of T5 in Eq. (8).

8 $$ Rutils\left( {\{ {\text{A}\} ,T}_{{5}} } \right) = {\text{I}}utils\left( {\{ C\} ,{\text{T}}_{5} } \right) = 1 \times 6 = 6 $$

The algorithm identifies a set whose TWU value exceeds the minimum utility threshold, and arranges it in ascending order during the initial database scan. During the second scan of the database, it is reorganized based on item set order, and a list of utilities is generated for each item set. Once the utility list of an item set has been generated, the algorithm combines the utility lists of {K − 1}-length to construct the utility list of {K}-length item sets. For instance, the utility list of {A, B} itemsets in Table 7 (the utility list of {A,B}) is constructed by merging the utility lists of {A} and {B} itemsets found in Table 8 (the utility list of {A} and {B}), as illustrated in Fig. 9 (TID = 6).

**Table 7:** The utility list of {A,B}.

Iutils	Rutils
14	12
32	9

**Table 8:** The utility list of {A} and {B}.

TID	Iutils	Rutils	TID	Iutils	Rutils
1	5	12	2	4	4
3	10	12	3	4	22
5	10	6	4	8	19
6	30	9	6	2	39

The FHM algorithm does not generate an effective set of candidate items. Instead, it employs estimated utility co-occurrence pruning (EUCP) to reduce the number of join operations. This technique is supported by the EUCS data structure, which is created during the second scan of the database. The EUCS structure determines whether the superset of any item set I is a High Utility Itemset (HUI) by checking whether the sum of *Iutils* and *Rutils* of the superset exceeds the minimum utility threshold. If it does not, the superset will not be further investigated, thus reducing the number of connections. Table 9 illustrates the EUCS structure of the database.

**Table 9:** Data structure of EUCS.

Item	A	B	C	D	E	F	G
B	80						
C	87	56					
D	97	110	90				
E	64	67	70	67			
F	37	37	0	37	37		
G	27	13	40	0	40	0	
H	43	43	43	43	0	0	0

### The IF-HUPM algorithm

The IF-HUPM main algorithm consists of three main modules. The first module is the Fuzzy function **Fuzzy( )**, which is responsible for fuzzifying the database^32,43^. The second module is the **UPGrowth( )** (Function 1), which employs a two-phase approach for mining fuzzy databases and incorporates a switching module. Then, the third module, **FHM( )** (Function 2), is called upon to compute the real utility values, compare them with the minimum utility threshold, and discover all the high-utility itemsets.The specific pseudo-code is shown in IF-FUPM Algorithm for the main function, and the **Up-Growth**(T, H, minUtil) is shown in Function 1.

**Figure:**
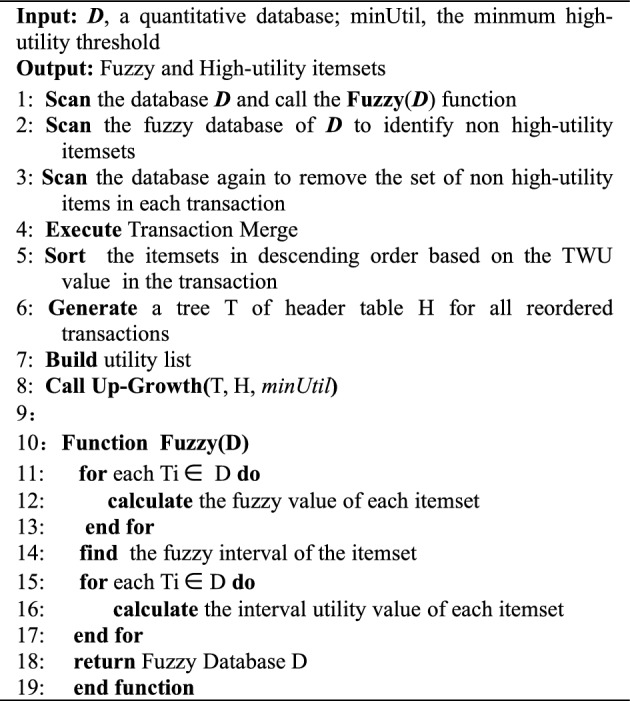
IF-FUPM Algorithm

**Figure:**
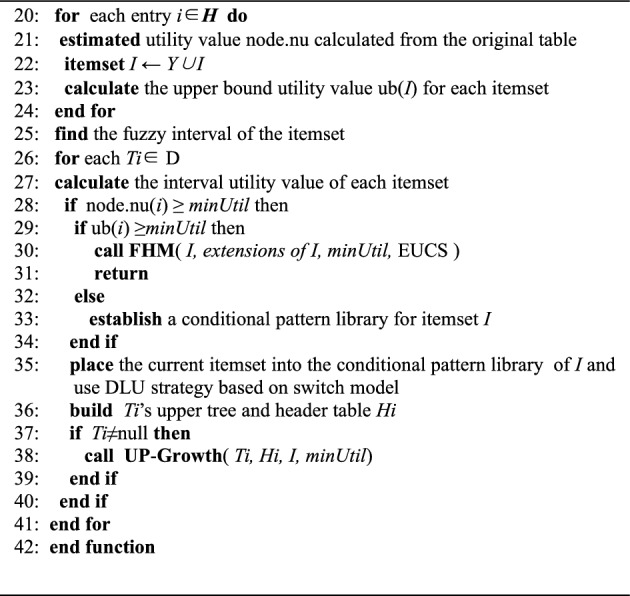
Function 1: Up-Growth(*T, H, minUtil*)

In the IF-FUPM Algorithm from line 1 to 8 involves scanning the original database, generating the fuzzy database through fuzzification, initializing data structures, and calling the Up-Growth( ) function. The pseudo-code from line 10 to 19 represents the fuzzy function module, which handles the fuzzification of the original database, computes frequent fuzzy intervals, and returns the fuzzy database.

**Figure:**
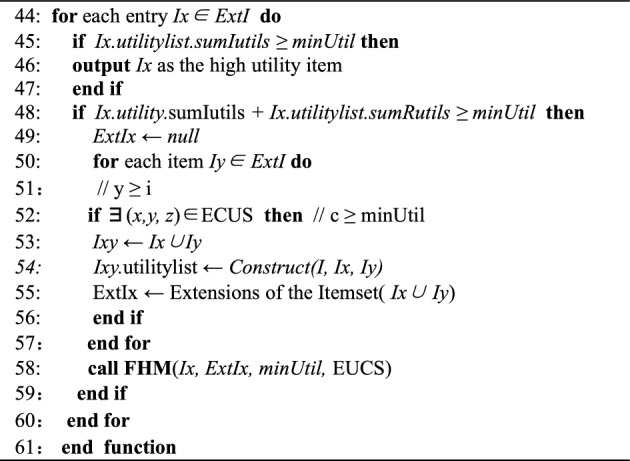
Funciton 2: FHM(*I, Extensions of I(ExtI), minUtil, EUCS*)

The pseudo-code for the UPGrowth( ) function is shown in Function 1, where lines 20–42 represent the two-phase mining process of the UPGrowth( ) of the fuzzy database. During the mining process, the algorithm calculates the overestimated utility values and compares them with the utility threshold to prune the utility tree. When a fuzzy high-utility 1-itemset is found, the algorithm checks whether the upper bound utility value is greater than or equal to the minimum utility threshold. If the condition is met, the FHM algorithm module is called, which is represented by lines 44 to 61 in Function 2. If not, the UPGrowth( ) function module will be called again until the switching criteria are met or all the fuzzy high-utility itemsets in the database are discovered.

The FHM( ) function is a one-phase high-utility itemset mining technique that directly computes real utility values and compares them with the user-defined utility threshold during execution. If a computed value exceeds the threshold, it is output, otherwise, it is discarded. Additionally, during the function's execution, the algorithm checks if all the fuzzy high-utility itemsets in the dataset have been discovered. If they have, the algorithm terminates and outputs the results of the mining, which are highly interpretable. Otherwise, it continues executing until all the fuzzy high-utility itemsets are found.

## Experimental evaluation

In this experiment, the algorithm presented in this paper is compared to the EFIM algorithm^53^ and UPHist algorithm^54^. To perform the comparison, several distinct datasets were used, including both publicly accessible real datasets and synthetic datasets, which are commonly used to evaluate data mining algorithms. One of the real datasets used in the experiment was the Diabetes dataset, which contains information on diabetes patients from the experimental database. The Pima dataset, made available to the public by the National Institute of Diabetes and Digestive and Kidney Diseases^55^, was also utilized. Additionally, the foodmart dataset, an actual customer transaction dataset for retail stores in SQL Server 2000, was used. Finally, the experiment included the Mushroom dataset, which is a synthetic dataset.

**Figure 11:**
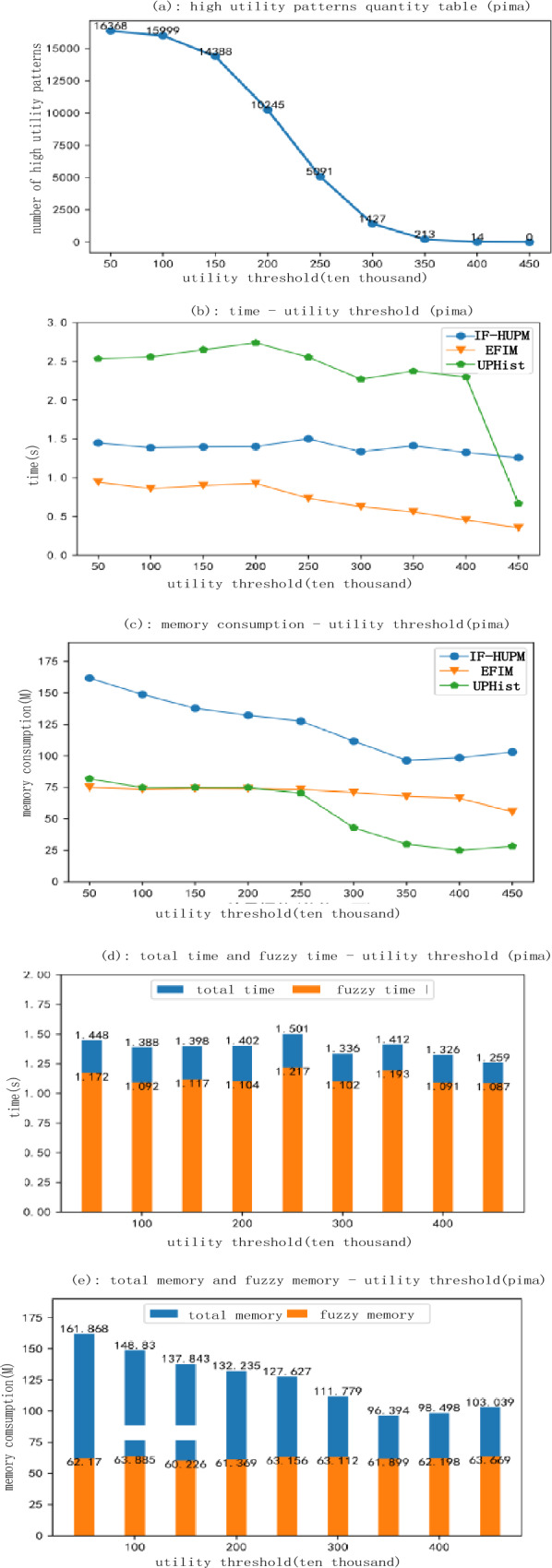
Experimental results for the Pima dataset.

**Figure 12:**
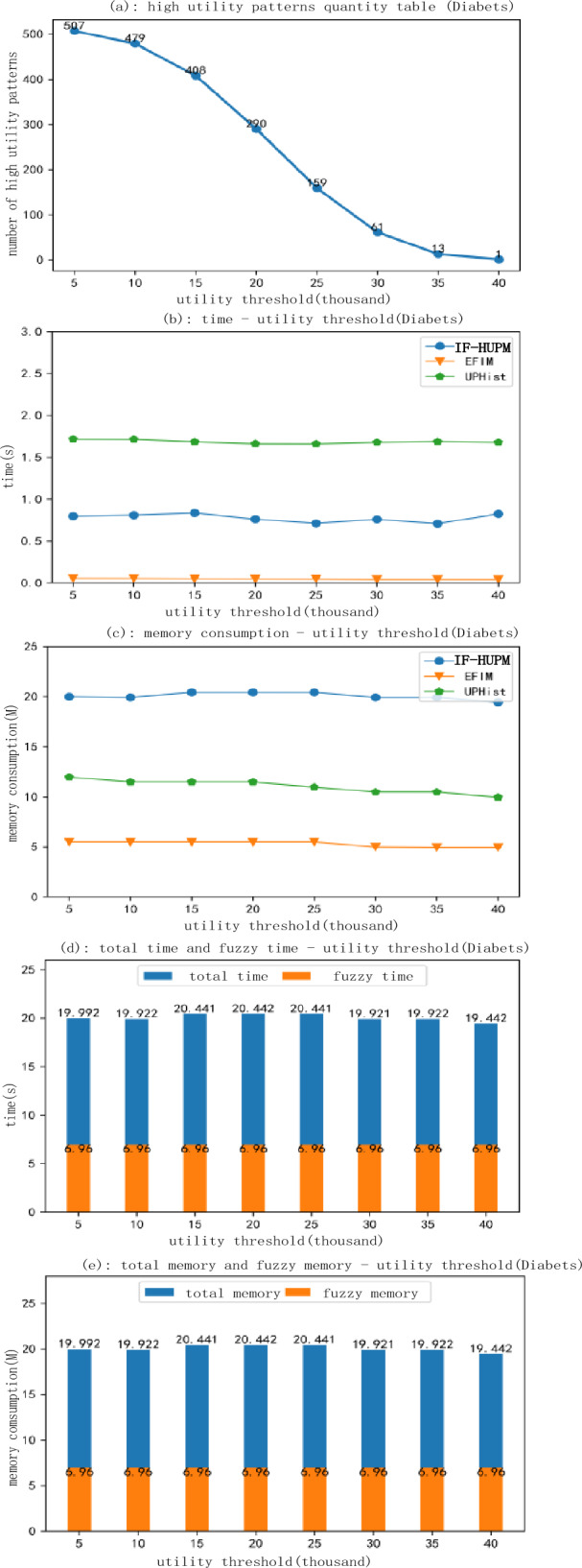
Experimental results for the diabetes dataset.

Table 10 shows the characteristics of the experimental dataset. The selected datasets are standard datasets with diverse characteristics, making them suitable for evaluating all aspects of the algorithm. The experimental results for each dataset are presented in Figs. 11 and 12.

**Table 10:** Data set feature table.

Name	Number	Number of items	Average length	Reality
Diabetes	58,968	14	14	Yes
Pima	768	9	9	Yes
Foodmart	4141	1559	4.42	Yes
Mushroom	8416	119	23	No

Throughout the execution of the algorithm, we assessed three distinct algorithms based on parameters such as runtime, memory usage, and the quantity of generated fuzzy high utility itemsets, all evaluated under varying utility thresholds. We conducted a comparative analysis across diverse datasets, identifying disparities amongst them as depicted in Figs. 11 and 12. Within an identical dataset, we instigated experiments by adjusting the utility threshold values incrementally from 500 to 4500 thousand at a fixed high utility value. Ultimately, we juxtaposed the performance of our proposed algorithm against EFIM and UPH, focusing on runtime and memory consumption.

Figure 11a illustrates a gradual decrease in the number of fuzzy high-utility itemsets mined in the Pima dataset as the minimum utility threshold ascends, with the rate of this decrease decelerating over time. Figure 11b indicates a relative stability in the execution time of the F-UFH, UPHist, and EFIM algorithms across varying utility thresholds. As depicted in Fig. 11c, memory consumption plateaus with an increase in the utility threshold. A review of the F-UFH model's experimental results in the Pima dataset (Fig. 11d) suggests a majority of processing time is allocated to the fuzzy process. Lastly, Fig. 11e reveals higher memory consumption during the fuzzy process than the mining process, although the total memory usage and distribution remain fairly constant.

Figure 12a illustrates a steady decrease in the number of discovered fuzzy high-utility itemsets in the Diabetes dataset with an increasing minimum utility threshold. Figure 12b reveals an initial slow decrease in runtime, accelerating in the latter half as the utility threshold increases, with notable performance improvement in the UPhist algorithm. Runtime drops significantly from 2.5 to about 0.8 s as the utility threshold increases from 4 to 4.5 million, surpassing the speed of F-UFH but lagging behind EFIM. As depicted in Fig. 12c, memory usage generally declines and stabilizes with utility threshold elevation, except for a minor surge in F-UFH at the maximum utility setting. Figure 12d exhibits the F-UFH experiments on the Diabetes dataset, revealing minimal time for fuzzification but high usage during the mining phase. Finally, Fig. 12e indicates constant memory usage in the mining process, yet decreasing memory consumption during fuzzification with an increasing minimum utility threshold, that ultimately stabilizes.

Evidently, the findings allow us to deduce definitive conclusions about temporal and spatial complexity. Reflecting on temporal complexity, an upward movement in the minimum utility threshold in the Diabetes dataset corresponds with a progressive decline in the quantity of high utility fuzzy itemsets detected. This illustrates the algorithm's enhanced pruning speed at higher utility thresholds, effectively diminishing the search space and thereby reducing temporal complexity. Notably, the UPhist algorithm demonstrates a significant time reduction from 2.5 to about 0.8 s when the utility threshold escalates from 4 to 4.5 million, signaling its high performance optimization at larger utility thresholds. Conversely, the F-UFH technique, while inferior in performance to UPhist, outperforms the EFIM algorithm at increased utility thresholds, underscoring the varying efficiency of different algorithms at different thresholds.

Exemplary performance is demonstrated by the algorithms concerning space complexity. With increasing utility thresholds, a decline and subsequent stabilization are observed in memory consumption, signaling the algorithms' commendable scalability in the realm of space complexity. One outlier to note is the increased memory usage of F-UFH at the maximum utility threshold, potentially due to the processing of a larger number of fuzzy itemsets at high thresholds. Referring to Fig. 12d, the application of the F-UFH model to the Diabetes dataset centers most processing time in the mining phase, with less time consumed in the fuzzification phase, indicating the high spatial complexity of the mining phase. Yet, Fig. 12e reveals consistent memory usage during the mining process with decreasing memory utilization during fuzzification, which ultimately stabilizes as the minimum utility threshold is enhanced. This underlines the algorithm's scalability in relation to space complexity.

Consolidating the aforementioned analysis leads us to infer that a rising minimum utility threshold corresponds to reduced temporal complexity, while spatial complexity commendably retains its scalability. Performance divergences are evident among algorithms interacting with different thresholds, thus underscoring the need to judiciously select algorithms congruent with specified scenarios in practical applications.

Table 11 presents a comparative evaluation of the IF-HUPM and FHTFUP^56,57^ algorithms in terms of runtime and memory usage. In our empirical assessment, we employed the Mushroom dataset. The comparison reveales that the IF-HUPM algorithm reported lower average temporal and spatial complexities as opposed to the FHTFUP algorithm. This quantitative data emphasizes the superior performance efficiency of IF-HUPM, signifying a balanced achievement between execution time and memory consumption.

**Table 11:** Results of statistical tests for performance.

Category	Algorithm	Mean value
Mushroom (4–16%)		
Runtime (s)	FHTFUP^56^	1826.148
	IF-HUPM	6.914
Memory (Mb)	FHTFUP^56^	128.6775
	IF-HUPM	90.0481

Besides, the final results of the algorithm proposed in this article are shown in the following Table 12. Each row represents a HUI, and the last column indicates the fuzzy utility value of that item set. Based on the first entry in Table 12, it can be inferred that based on the three interval of fuzzification(L represent Low, M represents Middle, H represents High based on Table 3). The BMI.H, FBG.H, HbAlc.H, ALT.L can form a HUI with a utility value of 1,147,576. When high-utility patterns occur frequently, interpretable fuzzy rules can be derived based on association rules. The high-utility fuzzy association rule that the corresponds to the most significant utility value when health indicators are poor can be explained. The fuzzy association rule can be presented like below for example, if BMI is High, FBG is High, HbAlc is High and ALT is Low, then health index is Low.

**Table 12:** The results of IF-HUMP algorithm.

BMI	FBG	HbA1c	ALT	SGOT	BP	TB	CB	SCr	BUN	TC	TG	LDL-C	HDL-C	Utility value
High	High	High	Low	–	–	–	–	–	–	–	–	–	–	1,147,576
High	High	High	Low	Low	–	–	–	–	–	–	–	–	–	1,388,186
High	High	High	Low	Low	Normal	–	–	–	–	–	–	–	–	1,666,380
High	High	High	Low	Low	Normal	Normal	–	–	–	–	–	–	–	1,953,729
High	High	High	Low	Low	Normal	Normal	High	–	–	–	–	–	–	2,164,514
High	High	High	Low	Low	Normal	Normal	High	Normal	–	–	–	–	–	2,398,469
High	High	High	Low	Low	Normal	Normal	High	Normal	Normal	–	–	–	–	2,778,447
High	High	High	Low	Low	Normal	Normal	High	Normal	Normal	Normal	–	–	–	3,105,209
High	High	High	Low	Low	Normal	Normal	High	Normal	Normal	Normal	Normal	–	–	3,466,534
High	High	High	Low	Low	Normal	Normal	High	Normal	Normal	Normal	Normal	Normal	–	3,736,776
High	High	High	Low	Low	Normal	Normal	High	Normal	Normal	Normal	Normal	Normal	High	41,988,899

*FBG* fasting blood glucose, *HbA1c* He Moglobin A1c, *ALT* alanine aminotransferase, *SGOT* serum glutamic oxaloacetic transaminase, *BP* blood protein, *TB* total bilirubin, *CB* conjugated bilirubin, *SCr* serum creatinine, *BUN* blood urea nitrogen, *TC* total cholesterol, *TG* triglyceride, *LDL-C* low density lipoprotein cholesterol, *HDL-C* high-density lipoprotein cholesterol.

## Conclusions and future research

We propose an innovative IF-HUIM algorithm for the medical field to overcome the difficulties in understanding and interpreting the HUPM algorithm. We have successfully solved this problem by fuzzifying the database and combining one-phase and two-phase algorithms. The results show that fuzzy processing exhibits relative stability in terms of time and space consumption even in the face of datasets with various features. Moreover, our mining process is more frugal in terms of average time and space requirements compared to EFIM and UPHist, thus demonstrating the efficiency of the algorithm.

Although the comprehensibility and interpretability of the algorithm is improved by fuzzifying the database, this may also lead to an increase in the complexity of the algorithm. In addition, the algorithm is primarily designed for medical data and may require further tuning and optimization for other types of data. This limitation makes the applicability of the algorithm in certain scenarios may be compromised. Nevertheless, our IF-HUIM algorithm still has significant advantages. It is innovative in that it improves the stability and efficiency of the algorithm through fuzzification and the combination of one-phase and two-phase algorithms. This is an important advantage because time and space efficiency are often key determinants of algorithm performance when dealing with large-scale datasets.

In addition, our algorithm shows superior performance when compared to other state-of-the-art algorithms. Compared to EFIM and UPHist, our mining process requires less time and space on average. This means that our algorithm requires fewer resources to process the same dataset and is therefore able to process more data. Our overall goal is to overcome the limitations of existing methods and further improve the effectiveness and applicability of the FHUPM algorithm so that it can better cope with the challenges of multidimensional medical data. Although the IF-HUIM algorithm still needs to be improved in some aspects, we believe that through continuous research and optimization, we have the ability to further improve the performance of the algorithm to better meet the needs of practical applications.

In the future, we plan to focus on the following three areas of research. First, we will explore the application of type-2 fuzzy set theory^58^ to the HUPM algorithm^59^ and compare it with the type-1 fuzzy set mining algorithm. This comparison aims to identify more valuable fuzzy high-utility itemsets in temporal pattern mining^1,8,9,60^. Second, we will investigate the interpretability of FHUPM and enhance its informativeness for various application scenarios, such as social network distribution^51^, recommendation in mobile app development^4^, anomaly detection^7^, and flow prediction^6^. Finally, building upon existing work, we aim to discover fuzzy association rules based on the fuzzy high utility pattern mining.

In conclusion, the utilization of data mining in the diagnosis and treatment of diabetes enables physicians to extract valuable insights from intricate medical datasets, identify potential association rules and patterns, and offer more precise and personalized diagnostic and treatment recommendations for patients with diabetes. This will significantly contribute to enhancing health management and optimizing treatment outcomes through improved interpretation of medical diagnostic decisions.

## Data Availability

The dataset used in this paper is a publicly available dataset sourced from the internet, and it can be accessed from the following website: https://www.kaggle.com/uciml/pima-indians-diabetes-database.

## Appendix: Glossary of terms

See Table

**Table 13:** Glossary of terms.

Nomenclature	Account for	Nomenclature	Account for
HUIM	High utility items mining	UFH	Hybrid algorithm based on UP-growth + and FHM
FIM	Frequent items mining	F-UFH	Fuzzy UFH
TWU	Transaction weighted utility	TID	Transaction ID
Iutils	Items utilities	Rutils	Residue utilities

13.
